# Anti-Migratory Effect of Vinflunine in Endothelial and Glioblastoma Cells Is Associated with Changes in EB1 C-Terminal Detyrosinated/Tyrosinated Status

**DOI:** 10.1371/journal.pone.0065694

**Published:** 2013-06-04

**Authors:** Amandine Rovini, Géraldine Gauthier, Raphaël Bergès, Anna Kruczynski, Diane Braguer, Stéphane Honoré

**Affiliations:** 1 Aix Marseille Université, Institut National de la Santé et de la Recherche Médicale UMR_S 911, Marseille, France; 2 APHM, Hôpital Timone, Marseille, France; 3 Centre de Recherche d'Oncologie Expérimentale, Institut de Recherche Pierre Fabre, Toulouse, France; University of Illinois at Chicago, United States of America

## Abstract

We previously showed that vinflunine, a microtubule-targeting drug of the *Vinca*-alkaloid family exerted its anti-angiogenic/anti-migratory activities through an increase in microtubule dynamics and an inhibition of microtubule targeting to adhesion sites. Such effect was associated with a reduction of EB1 comet length at microtubule (+) ends. In this work we first showed that the pro-angiogenic vascular endothelial growth factor VEGF suppressed microtubule dynamics in living Human Umbilical Vein Endothelial Cells (HUVECs), increased EB1 comet length by 40%, and induced EB1 to bind all along the microtubules, without modifying its expression level. Such microtubule (+) end stabilization occurred close to the plasma membrane in the vicinity of focal adhesion as shown by TIRF microscopy experiments. Vinflunine completely abolished the effect of VEGF on EB1 comets. Interestingly, we found a correlation between the reduction of EB1 comet length by vinflunine and the inhibition of cell migration. By using 2D gel electrophoresis we demonstrated for the first time that EB1 underwent several post-translational modifications in endothelial and tumor cells. Particularly, the C-terminal EEY sequence was poorly detectable in control and VEGF-treated HUVECs suggesting the existence of a non-tyrosinated form of EB1. By using specific antibodies that specifically recognized and discriminated the native tyrosinated form of EB1 and a putative C-terminal detyrosinated form, we showed that a detyrosinated form of EB1 exists in HUVECs and tumor cells. Interestingly, vinflunine decreased the level of the detyrosinated form and increased the native tyrosinated form of EB1. Using 3-L-Nitrotyrosine incorporation experiments, we concluded that the EB1 C-terminal modifications result from a detyrosination/retyrosination cycle as described for tubulin.

Altogether, our results show that vinflunine inhibits endothelial cell migration through an alteration of EB1 comet length and EB1 detyrosination/retyrosination cycle.

## Introduction

Cell migration plays a crucial role in neoangiogenesis and tumor progression. Major components of the cytoskeleton, microtubules (MT) are key players involved in cell polarization and migration in many cell types such as fibroblasts and endothelial cells [1], [2]. MT are highly dynamic polymers that alternate between periods of growth and shortening, intervened by phases of no detectable activity. During cell migration, stabilized MT are selectively located at the leading edge and show a specific enrichment in tubulin post-translational modifications such as detyrosinated tubulin [3]. Recently, several works have revealed the crucial role of the (+) end tracking protein EB1 in cell migration [4], [5], [6]. EB1 belongs to a conserved family of proteins, the +TIPs, that specifically track MT (+) ends and regulate MT dynamics [7], [8], [9], [10]. EB1 is now considered as the master regulator at MT (+) ends, since it has been shown to act as a loading factor of other MT-interacting proteins, including those responsible for the MT stabilization at the cell cortex [11], [12]. Indeed, the hallmark of +TIPs is that they form dynamic interaction networks that rely on a limited number of protein modules and linear sequence motifs, such as the CH, EBH and CAP-Gly domains, and EEY/F and SxIP motifs [11]. For example, EB1 C-terminal sequence −EEY is responsible for interaction with the CAP-Gly domain containing +TIPs such as CLIP-170 and p150^Glued^, and their localization at MT (+) ends [9]. Importantly, EB1 shares with tubulin the same C-terminal sequence −EEY that undergoes a detyrosination/retyrosination cycle under the action of a tubulin carboxypeptidase and the tubulin tyrosine ligase, respectively. Tyrosinated tubulin is a crucial factor for the recruitment of CLIP-170 to MT [13]. To date, no post-translational modification of the C-terminal extremity of EB1 has been identified.

MT are important targets for cancer chemotherapeutic drugs, such as *Vinca*-alkaloids and taxanes. These MT targeting agents (MTAs), which suppress MT dynamics at cytotoxic concentrations [14], [15], are extensively used for treatment of various human malignancies. Vinflunine (VFL), latest MTA of the *Vinca*-alkaloid family, has been approved in monotherapy by the European MEdicine Agency (EMEA) for the treatment of adults with advanced or metastatic urothelial cancers. Anti-angiogenic, anti-vascular and anti-metastatic properties of vinflunine have been shown *in vitro* and *in vivo* [16]. Some potential underlying mechanisms of the anti-angiogenic property of MTAs have been reviewed [17], [18]. Interestingly, we have shown that MTAs, including paclitaxel and VFL, may produce their anti-migratory/anti-angiogenic effects through an increase in interphase MT dynamics in endothelial cells [16], [19]. Such effect was associated with a decrease of MT stabilization at the cell cortex, and an inhibition of MT targeting to adhesion sites, leading to the formation of long-lived stress fibers. Importantly, these effects were associated with an inhibition of EB1 accumulation at MT (+) ends, as shown by the decrease of EB1 comet length [20]. Recently, we also demonstrated that the anti-migratory action of the MT stabilizing drug patupilone occurred through an alteration of EB1 accumulation at MT (+) ends in glioblastoma cells [21].

In the current study we further investigated the role of EB1 under factors, namely Vascular Endothelial Growth Factor (VEGF) and VFL, that modulate cancer progression and in particular the migration process. We first showed that the pro-angiogenic/pro-migratory VEGF suppressed MT dynamics in HUVECs, and induced MT (+) end stabilization in the vicinity of focal adhesions. Moreover, VEGF increased EB1 comets length and induced EB1 to bind along the MT lattice, effects that were completely abolished in the presence of VFL.

Hypothesizing that VEGF and VFL could modulate EB1 at a post-translational level, we demonstrated for the first time, by 2D electrophoresis, that EB1 underwent post-translational modifications. Interestingly, we found that EB1 was detyrosinated in endothelial and glioblastoma cells. VFL decreased the detyrosinated form and increased the native tyrosinated form of EB1 in a concentration-dependent manner. By using 3-L-Nitrotyrosine incorporation experiments, we concluded that EB1 C-terminal post-translational modifications may result from a detyrosination/retyrosination cycle as described for tubulin. Altogether, our results show that VFL inhibits endothelial cell migration through an alteration of EB1 comet length and EB1 detyrosination/retyrosination cycle

## Materials and Methods

### Antibodies and reagents

We used anti-EB1 clone 5, anti-p150^Glued^ (BD Biosciences); anti-nitrotyrosine (Invitrogen); anti-YL ½ (Millipore); anti-detyrosinated EB1 and anti-tyrosinated EB1 are kind from gifts from Dr M.J Moutin and Dr A. Andrieux [22]. Secondary antibodies were HRP-conjugated donkey anti-mouse, anti-rabbit, and anti-guinea pig (Jackson ImmunoResearch Laboratories, Inc.); TRITC-, FITC-, anti-mouse and anti-rabbit (Jackson ImmunoResearch Laboratories, Inc.).

We used VEGF, gelatin, fibronectin, 3-Nitro-L-tyrosine and 3-(4,5-dimethylthiazol-2-yl)-2,5-diphenyltetrazolium bromide (MTT) assay, VEGF-Trap: VEGF receptor 2 (Flk-1; KDR)/Fc Chimera/human was used at 100 ng/ml for all experiments (Sigma-Aldrich); ascorbic acid (Aguettant).

A stock solution of vinflunine (Pierre Fabre Oncology) was prepared in distilled water and maintained frozen at −20°C.

### Cell culture and drugs

Human umbilical vein endothelial cells (HUVECs) were obtained from the Cell Culture Laboratory in the “Hôpital de la Conception”, Assistance Publique-Hôpitaux de Marseille; human glioblastoma (U87MG) cell line was purchased at the ATCC (Manassas, VA, USA). HUVECs were grown in EGM2 supplemented with single quots (Lonza) whereas U87 cells were grown in EMEM media supplemented with 10% FBS, 2% L-glutamine, 1% penicillin and streptomycin until reaching 80–90% confluence [19], [23]. HUVECs were used at passage 2–5, seeded on gelatin (2%) or fibronectin (10 µg/ml) coated flasks. Cells were routinely maintained in culture at 37°C and 5% CO_2_ in a humidified environment during culture and imaging.

For experiments on EB1 cycle of detyrosination/tyrosination, 3-Nitro-L-tyrosine (Sigma-Aldrich) was added to cell medium at a final concentration of 500 µM during 48 h and medium was renewed every 24 h.

### Transfection of U87 cells for migration assay

shRNA plasmid that specifically knocked out human EB1 (NM_012325) and negative shRNA control plasmid (Mission® non-target shRNA control vector) were obtained from Sigma-Aldrich. U87 cells at 80% confluence were transfected with lipofectamine™ 2000 system (Invitrogen) according to the manufacturer's instructions. For establishing stable clones, the transfected cells were selected in EMEM 1640 medium containing puromycin (Sigma-Aldrich) at 0.5 µg/ml 24 h post-transfection. After western blot analysis, U87 clone under-expressing EB1 and negative control-transfectants were selected as U87 shEB1-clone 11 (U87 sh11) cells and (U87 sh0) respectively.

For transient transfection, GFP-EB1 full length, GFP-EB1ΔY expression plasmids and GFP negative control plasmid were kind gifts from Dr Holly Goodson (University of Notre Dame, IN, USA). U87 cells at 80% confluence were transfected with lipofectamine™ 2000 system according to the manufacturer's instructions. 24 h post-transfection, cells were analyzed for protein expression by western blot and used for *in vitro* assays.

### Cytotoxicity assay

Cells (5000 cells/well) were seeded in 96-well plates and allowed to grow for 24 h before treatment with VFL. Growth inhibition of HUVECs was measured after 72 h by using the MTT cell viability assay, as previously described [16].

### Cell migration experiments

#### 2D random cell motility

HUVECs (20 000 cells/well) were seeded in 6-well plates and were imaged with Nikon TE 2000 microscope (Nikon) equipped with a digital camera (CCD camera coolsnap HQ, Princeton Instruments) at low magnification (×10) for 5 h. Conditions tested include cells treated with VEGF (10 ng/ml) or pre-incubated with VEGF Trap (30 min, 100 ng/ml). Random motility coefficient was calculated as previously described [20].

#### In vitro wounding-healing assay

HUVECs (500 000 cells/well) were seeded in 6-well plates and allowed to grow for 24 h before being wounded with a tip. Image acquisition of cell migration was made each 10 min during 15 h. Four fields per filter at a magnification of 10× were imaged and quantification of surface recovery was made with Metamorph software®.

#### Transwells

HUVECs or U87 (50.000 per condition) were poured on the upper side of a transwell migration chamber (0.8 µm filter, BD) in serum free medium. The lower side of the chamber was filled with culture medium completed with VEGF (10 ng/ml) for migration of HUVECs or standard culture medium for U87 cells. Cells were allowed to transmigrate for 5 h and then chambers were removed. Cells that did not migrate stayed on the upper part of the filter and were removed with a cotton stick; cells on the lower side of the filter were fixed with 1% glutaraldehyde (Sigma- Aldrich) and stained with 1% Crystal-violet solution in 20% methanol. After washing and drying, pictures of four fields per filter were taken at a magnification of 10×. Quantification of cell transmigration was made with Metamorph software ® and results were expressed as percent of cells that transmigrated (mean ±SEM). More than three independent experiments were performed.

### Time-lapse microscopy and analysis of microtubule dynamics

To analyze MT dynamics, HUVECs were transfected by nucleofection, according to the manufacturer instructions (Amaxa system, Lonza). Briefly, 5 µg of a plasmid coding for green fluorescent protein GFP–**α**-tubulin (Clontech Laboratories) was added to the cell suspension, which was transferred to a 2.0 mm electroporation cuvette and nucleofected (Nucleofector, Amaxa). After transfection, cells were immediately plated in complete medium. 24 h later, cells were treated for 1 h with VEGF at 10 ng/ml or VEGF Trap at 100 ng/ml, and time-lapse microscopy analysis was done.

To measure MT dynamics, GFP–**α**-tubulin-transfected cells were placed in a double coverslip chamber maintained at 37°C, in EGM2 culture medium supplemented with ascorbic acid (0.1 mg/mL) and analysis of MT dynamics was done as described previously [6], [16], [20]. Using the track point function of the Metamorph software®, changes in length >0.5 µm were considered as growth or shortening events and changes <0.5 µm were considered as phases of attenuated dynamics or pauses. The rates of growth and shortening events were determined by linear regression. Means and SEM were calculated per event. The catastrophe frequency based on time was calculated by dividing the number of transitions from growth or pause to shortening by the total time growing and paused for each individual microtubule. The rescue frequency based on time was calculated similarly, dividing the total number of transitions from shortening to pause or growth by the time spent shortening for each individual microtubule. Means and SEM of transition frequencies were calculated per microtubule. Overall dynamicity was calculated as the total length of growth and shortening divided by MT population life span.

### Indirect immunofluorescence analysis

Cells were fixed either for 5 min with cold methanol (−20°C) or 3,7% formaldehyde and stained as described previously for EB1 [24] or with p150^Glued^(1/100). Samples were mounted with ProLong-Gold antifade reagent (Invitrogen).

EB1 comets measurements were realized as described [24] and values are expressed as mean ± SD.

### Western Blot analysis and 2D Gel electrophoresis

#### Isoelectric focusing

Isoelectric focusing was performed with 18 cm Immobiline DryStrips pH 4–7, (GE Healthcare). A total amount of 100 µg of protein cell lysate in IPG buffer (8 M urea, 2 M thiourea, 4% CHAPS, 0.5% TX-100, 0.5% ampholytes (pH 4–7), 20 mM DTT and a few grains bromphenol Blue) was loaded on the IPG strip using Ettan IPGphor 2 (Amersham Biosciences) with a focusing time of 21 h (27 kVh total).

#### 2D-electrophoresis

Prior to second-dimension, IPG strips were incubated with Tris acetate equilibration buffer (75 mM Tris, 6 M urea, 30% glycerol, 0.25% DTT) for 15 min. Following this reduction step, alkylation with 2.5% iodoacetamide in 75 mM Tris, 6 M urea, 30% glycerol was performed for 15 min. The IPG strip were then placed on the top of a 4–20% Criterion precast gel (Bio-Rad) and run for 1.5 h at a constant 150 V in a Criterion Dodeca Cell tank (Bio-Rad) using a standard Tris-glycine SDS running buffer at 12°C. Then, SDS-PAGE electrophoresis was performed using 4–20% precast Tris-HCl Gels (Bio-Rad) and the Bio-Rad Mini-Protean gel electrophoresis system; immunoblot analysis was performed by electroblotting SDS-PAGE-resolved proteins onto transfer nitrocellulose membrane (Amersham Biosciences). All experiments were done in triplicate.

#### Western blotting

For 1D gel electrophoresis cells were lysed (lysis buffer: Tris 50 mM pH 8.0, NaCl 250 mM Triton-x100 1%; SDS 0.1%) and 30 to 100 µg of total protein lysate were loaded onto a 12% SDS-PAGE gel. Western blot was performed as previously described [23]. Experimental isoelectric point for each spot was determined according to reference proteins such as stathmin and theoretical isoelectric points of EB1 post-translational modifications were calculated by using Expasy software®.

## Results

### VEGF -induced endothelial cell migration was associated with suppression of microtubule dynamics

We previously showed that anti-angiogenic concentrations of VFL increased MT dynamics instability in living HMEC-1 cells and disrupted microtubule-focal adhesion crosstalk [16], [20]. Here, we first showed that VEGF (10 ng/ml) enhanced HUVECs migration into both a 2D random cell motility experiment and wound healing assay (Figure S1). Then, we analyzed the effect of VEGF on MT dynamics by tracking GFP-α-tubulin transfected HUVECs. We observed that VEGF (10 ng/ml) led to an inhibition of MT overall dynamicity from 4.9 µm/min in control cells to 2.4 µm/min in VEGF-treated cells, characterized by a decrease in the MT growth rate (11.3 µm/min in control cells to 8.4 µm/min in VEGF-treated cells, p<0.001) and in the shortening rate (14.1 µm/min in control cells to 10.2 µm/min, p<0.001), the percentage of time spent growing (3,2 *vs* 12,7%), and increased the duration of MT pauses (0.27 min in control cells to 0.45 min in VEGF-treated cells, p<0.001) (Table 1). In addition, blocking the autocrine VEGF effect on HUVECs by using VEGF Trap led to a significant increase in overall MT dynamicity from 4.9 µm/min in control cells to 7.0 µm/min in VEGF Trap-treated cells accompanied by a significant increase in both MT growth rate (11.3±0.7 µm/min *vs* 14.3±1.2 µm/min, p<0.05) and shortening rate (14.1±1.0 µm/min *vs* 20.2±1.3 µm/min, p<0.001), and also in the transition frequencies, i.e catastrophes (2.1±0.3 m^−1^ *vs* 1.8±0.3 m^−1^, p<0.05) and rescues (5.6±0.8 m^−*1*^ *vs* 4.2±0.8 m^−1^, p<0.01) (Table 1).

**Table 1:** Effect of VEGF and VEGF-Trap on MT dynamic instability parameters in HUVECs.

Parameters	Control	VEGF (% change/Control)	VEGF Trap (% change/Control)
Number of analyzed MT	29	43	48
Growth Rate (µm/min ± SEM )	11.3±0.7	**8.4±0.6** ***	**14.3±1.2** *
Shortening Rate (µm/min ± SEM )	14.1±1.0	**10.2±0.5** ***	**20.2±1.3** ***
Pause duration (min ± SEM)	0.27±0.03	**0.45±0.03** ***	0.26±0.02
%Time spent Growing	12.7	3.2	15.6
% Time spent Shortening	24.8	20.6	23.8
% Time spent in Pause	62.5	76.2	60.6
Catastrophe (min ^−1^± SE )	1.8±0.3	1.5±0.3	**2.1±0.3** *
Rescue (min ^−1^± SE)	4.2±0.8	4.5±0.7	**5.6±0.8** **
Overall dynamicity (µm/min)	**4.9**	**2.4**	**7.0**

* p<0.05;

** p<0.01;

*** p<0.001 (vs control); ns: not statistically significant.

Moreover, in HUVECs co-transfected with GFP-tubulin and DsRed-paxillin, we showed that MT (+) ends localized close to the plasma membrane in control and VEGF treated cells as observed by TIRF microscopy (Figure S2A). In addition, VEGF increased MT targeting to adhesion sites (Figure S2 B, C). Altogether, these results indicate that VEGF suppressed MT dynamic instability and increased MT targeting to adhesion sites.

### VEGF increased EB1 accumulation at microtubule (+) ends

**Figure 1:**
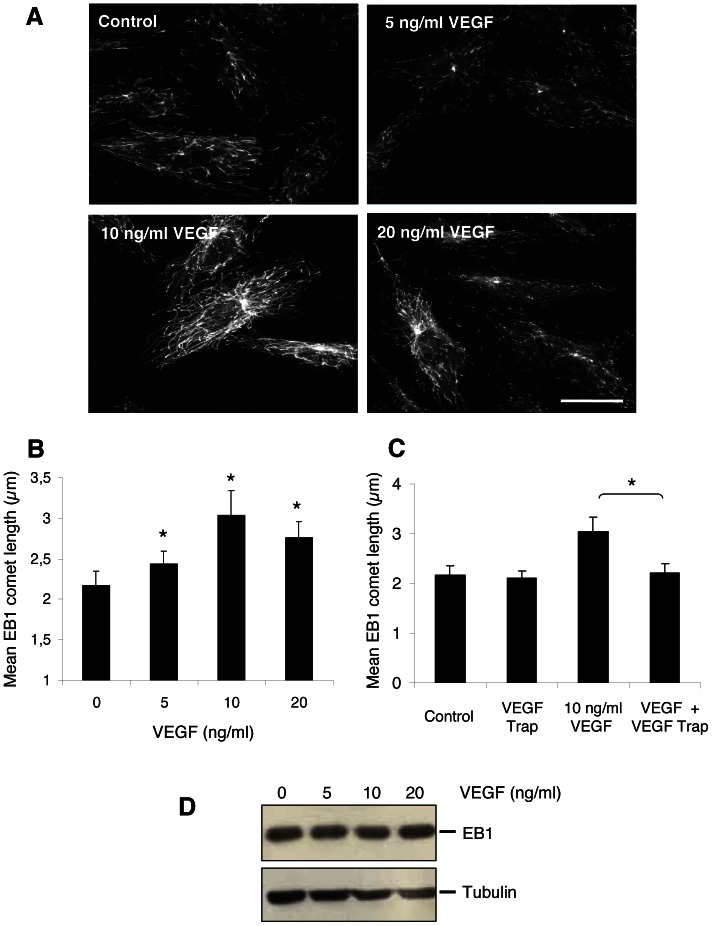
Effect of VEGF on EB1 comets. (**A**) Localization of EB1 in HUVECs by indirect immunofluorescence after 1 h of VEGF treatment at various concentrations. *Bar*, 10 µm. (**B**) Dose response effect of VEGF on EB1 comet length. * p≤0.001 (vs control, Student *t* test). (**C**) Incubation of HUVECs with VEGF trap (1 h) inhibited VEGF-increase of EB1 comet length. * p≤0.001. (**D**) Western blotting detection of EB1 and tubulin in HUVECs after 1 h of treatment with VEGF at various concentrations.

We then explored the effects of VEGF on EB1 comet length by indirect immunofluorescence (Figure 1). At 10 ng/ml VEGF increased EB1 comet length by 40% (3.03±0.3 µm vs 2.17±0.18 µm, p<0.05 (Figure 1 A, B) and induced, in nearly 20% of cells, a complete MT lattice staining (Figure 1A). HUVECs pre-incubation with VEGF-Trap inhibited VEGF increase of EB1 comets, suggesting that such effect was specific of VEGF signaling (Figure 1C). Interestingly, the modification of EB1 comet length by VEGF was not associated with any change in EB1 expression level (Figure 1D).

Altogether these results indicate that exogenous VEGF stabilized microtubules at the cell periphery, while increasing EB1 comet length and lattice binding.

### Pro-migratory VEGF induced EB1 C-terminal modifications

In order to decipher the mechanisms by which VEGF altered EB1localization on microtubules, we chose to investigate whether it could induce post-translational modifications, as far as very little is known on such regulation. By using 2D-electrophoresis, we first showed that EB1 focalized in several isoelectric points ranging from 4.86 to 5.28 (Figure S3). The two main spots of EB1 focalized at the 5.02 which corresponds to the theoretical isoelectric value of the native form and a spot at 4.95 which corresponds to the theoretical isoelectric value of one site phosphorylated EB1 (Expasy®). Similar 2D profiles of EB1 were found in U87 glioblastoma cells (Figure S3), demonstrating that EB1 post-translational modifications were not restricted to HUVECs.

**Figure 2:**
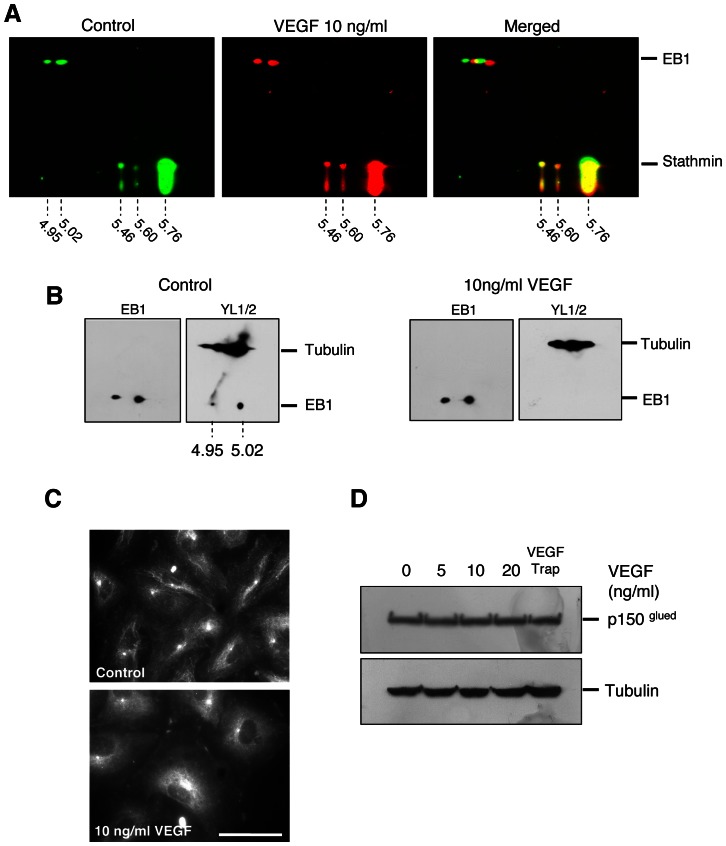
VEGF induced EB1 C-terminal −EEY modification and loss of CAP-Gly protein interaction. (**A**) Effect of VEGF on EB1 focalization on 2D gel electrophoresis. Cells were incubated for 1 h with VEGF (10 ng/ml) or vehicle alone. Indicated experimental isoelectric points were calculated according to reference protein (stathmin). (**B**) Detection of −EEY sequence with YL½ antibody by 2D gel electrophoresis of HUVECs under control and VEGF (10 ng/ml) treatment. YL½ antibody allowed the detection of the −EEY sequence both in tubulin and EB1. Revelation of EB1 using anti-EB1 antibody on the same membrane after stripping is presented for each condition ( left of panels). (**C**) Localization of p150^Glued^ in HUVECs treated or not with VEGF (10 ng/ml) by indirect immunofluorescence. *Bar* = 10 µm. (**D**) Western blotting detection of p150^Glued^ and tubulin in HUVECs after 1 h of treatment with VEGF at various concentrations or VEGF Trap.

We then analyzed the influence of VEGF treatment on the EB1 2D profile as compared to control cells. Interestingly, by using stathmin as an internal reference, we found that VEGF induced a 0.01 pH unit shift of the two main spots of EB1 toward a more basic form (Figure 2A).

To further precise the changes induced by VEGF,, we used the YL½ antibody, which specifically recognizes the C-terminal –EEY epitope shared by tubulin and EB1 [25], [26]. Indeed, in control cells the YL½ antibody recognized both the two main spots of EB1 and, as expected, tubulin. Interestingly, in cells treated by VEGF, the YL½ antibody failed to recognize the –EEY epitope on EB1 while it recognized the tubulin –EEY epitope in the same gel (Figure 2B).

To evaluate the consequence of VEGF-induced EB1 C-terminus modification on interaction with its partners, we investigated whether the CAP-Gly protein p150^Glued^, which is known to interact with the –EEY sequence of EB1 and tubulin [27], [28], [29], [30], still binds to EB1-decorated MT (+) ends. Consistent with the alteration of EB1-EEY sequence under VEGF treatment, p150^Glued^ which decorated MT (+) ends in control cells, disappeared in VEGF treated cells (Figure 2C). As for EB1, VEGF treatment did not modify p150^Glued^ expression level (Figure 2D).Taken together these results showed that VEGF induced an alteration of CAP-Gly protein binding to EB1 in link with an alteration of the EB1-EEY motif.

### A detyrosinated form of EB1 exists in human endothelial and glioblastoma cells

**Figure 3:**
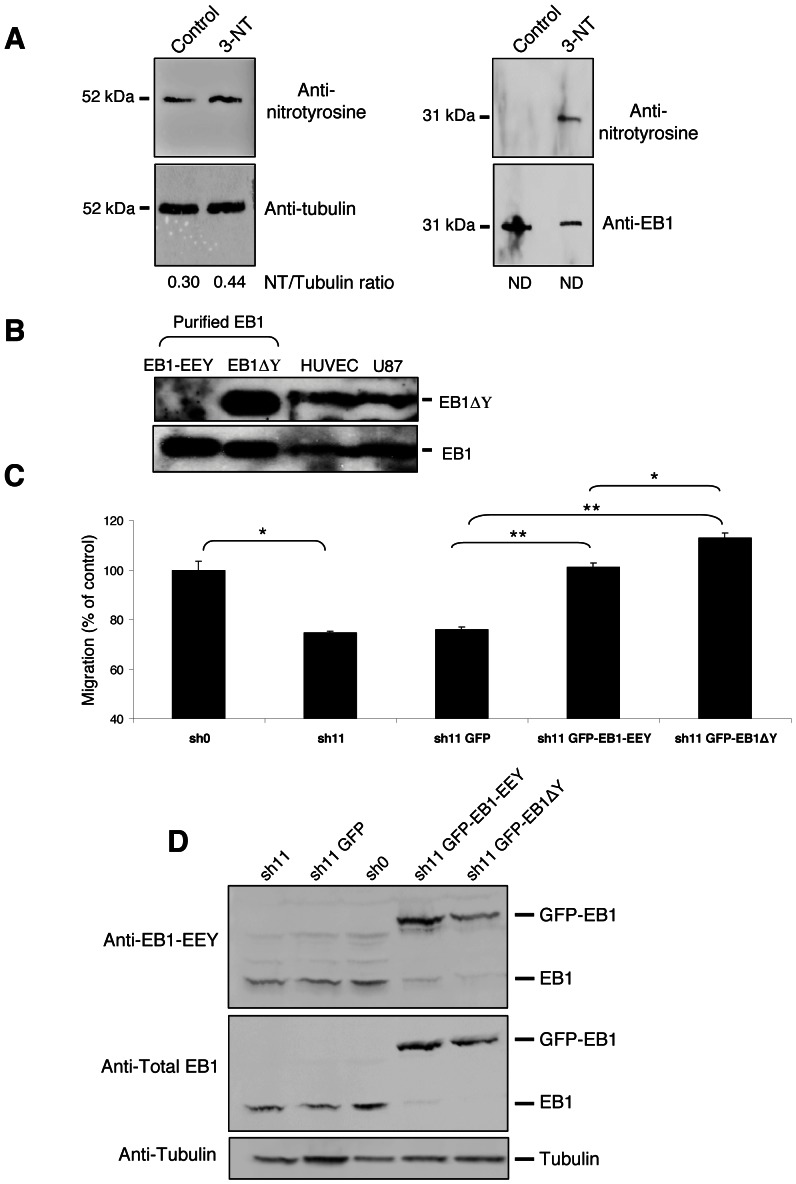
Detection of detyrosinated EB1 in HUVECs and U87 cells. (**A**) HUVECs incubation with 3-L-nitrotyrosine allowed detection, in total cell lysates, of a nitrotyrosinated form of tubulin but also of EB1. Representative of 3 independent experiments (**B**) Purified tyrosinated and detyrosinated EB1 (EB1-EEY, EB1ΔY) proteins were loaded as, respectively, negative and positive probes in regard to detyrosinated EB1 detection in HUVECS and U87 total cell lysate by using a specific guinea pig anti-detyrosinated EB1 antibody [22] (**C**) Transwell migration assay was performed in U87 cells depleted of EB1 (clone sh11) and expressing GFP-EB1 full length or GFP-EB1ΔY mutant. U87 sh0 clone was also used as a negative control. Data show the average number of cells that migrated. At least three independent experiments were performed for each condition. *Bar* ± S.E.M. (*) indicates significant differences from controls: *, p<0.05; **, p<0.005. (**D**) Western blot was performed with down-regulating EB1 U87 sh11 clone transfected or not with GFP-EB1 full length, GFP-EB1ΔY expression plasmids or control GFP-empty vector. U87 sh0 clone was also used as a negative control. Tyrosinated-EB1 was detected by using a specific anti-EB1 tyrosinated antibody [22].

According to the requirement of the last tyrosine residue for EB1/CAP-Gly interaction [29] and the similarity in the C-terminal –EEY sequence between α-tubulin and EB1, one can expect to find the same post-translational modifications to be generated and notably a cycle of detyrosination/retyrosination. HUVECs incubation with 3-L-nitrotyrosine, a non-genetically coded amino acid, during 48 h resulted in its incorporation in tubulin, as expected (Figure 3A) [31], [32]. Interestingly, we found that EB1 also incorporated 3-L-nitrotyrosine (Figure 3A). As this result led to suspect the existence of a detyrosinated form of EB1, we tested its existence in both endothelial and glioblastoma cells by using a specific antibody against the detyrosinated form of EB1 [22]. This antibody that failed to recognize purified tyrosinated EB1 (Figure 3B), detected a detyrosinated form of EB1 in HUVEC and U87 cell lysates (Figure 3B).

We then evaluated the functional consequence of such detyrosinated form of EB1 in terms of cell migration. For this purpose, we performed rescue experiments in EB1 shRNA down-regulated U87 clones using GFP-EB1 full-length or GFP-EB1ΔY constructs. Down-regulation of EB1 significantly decreased cell migration by 25.5% (p<0.05). Re-introduction of both GFP-EB1 full length and GFP-EB1ΔY significantly rescued U87 cell migration (p<0.01) (Figure 3C). Interestingly, we found that GFP-EB1ΔY more significantly rescued cell migration as compared to GFP-EB1 full-length construct (+17%; p<0.05). In addition, we observed that GFP-EB1ΔY expression was lower than GFP-EB1 full-length expression (Figure 3D).

Moreover, we noticed that the GFP-EB1ΔY fusion protein was partly retyrosinated suggesting that the retyrosination process also occurred in glioblastoma cells.

### Inhibition of HUVEC migration by vinflunine was correlated with EB1 comet disruption

**Figure 4:**
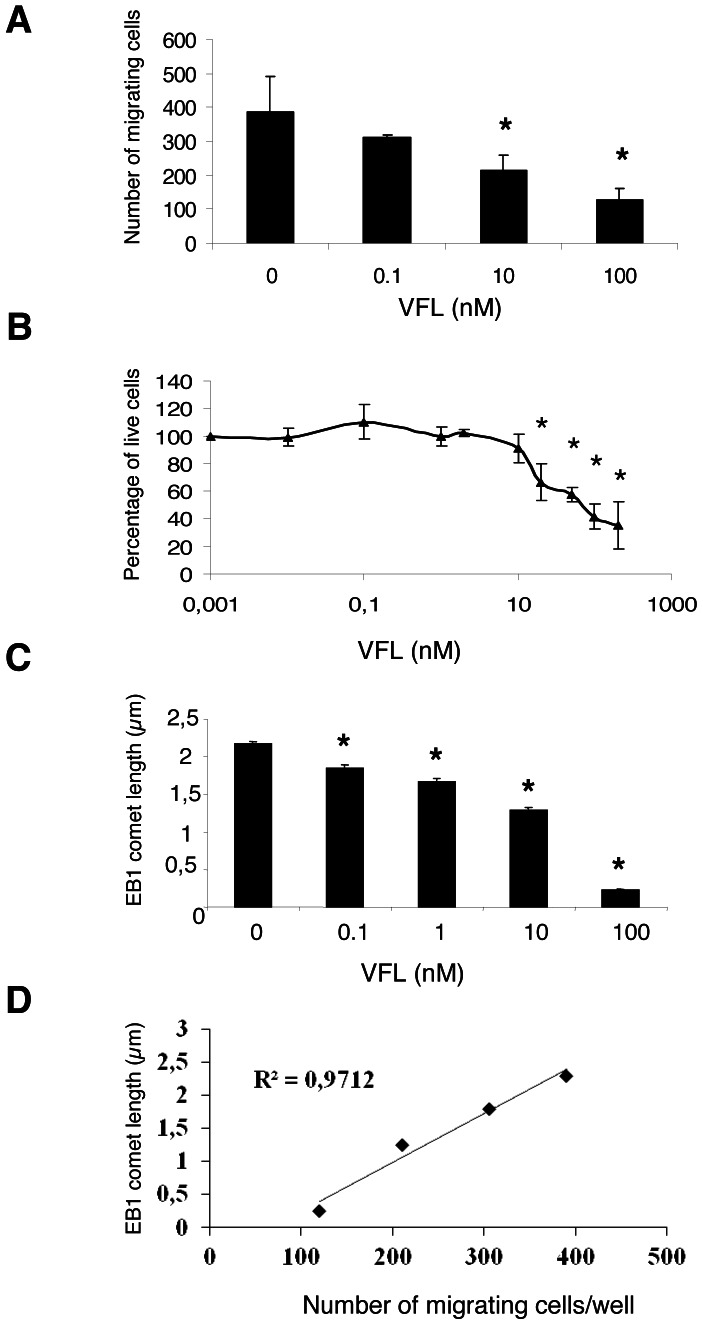
Anti-migratory vinflunine decreased EB1 comet length. (**A**) Number of migrating HUVECs in transwells following VFL treatment. *p≤0.001 (*vs* control, Student *t* test). (B) Concentration-dependent inhibition of cell survival by VFL after 72 h of treatment (**C**) Dose-response effect of VFL on EB1 comet length. * p≤0.05 (*vs* control, Student *t* test). (**D**) Correlation between EB1 comet length and the mean number of migrating cells/well.

We previously demonstrated that inhibition of U87 and HMEC-1 migration was correlated with EB1 comet length reduction [21], [22]. Here we explored VFL dose-response effect on endothelial cell migration and EB1 comet. In HUVECs, VFL suppressed migration in a concentration-dependent manner from 0.1 nM to 100 nM (Figure 4A, p<0.05) while concentrations required to inhibit cell viability were over 10 nM (Figure 4B). Moreover, VFL decreased EB1 comet length in a concentration-dependent manner, with a nearly complete disruption of EB1 at MT(+) ends at 100 nM (Figure 4C). Interestingly, we evidenced a strong correlation between the reduction of EB1 comet length and cell migration inhibition suggesting that such effect may contribute to the anti-migratory activity of VFL (Figure 4D). Altogether, our data suggest that the anti-migratory effect of VFL is correlated to EB1 comet disruption.

### Anti-migratory concentrations of vinflunine favored EB1 tyrosinated form in both endothelial and glioblastoma cells

**Figure 5:**
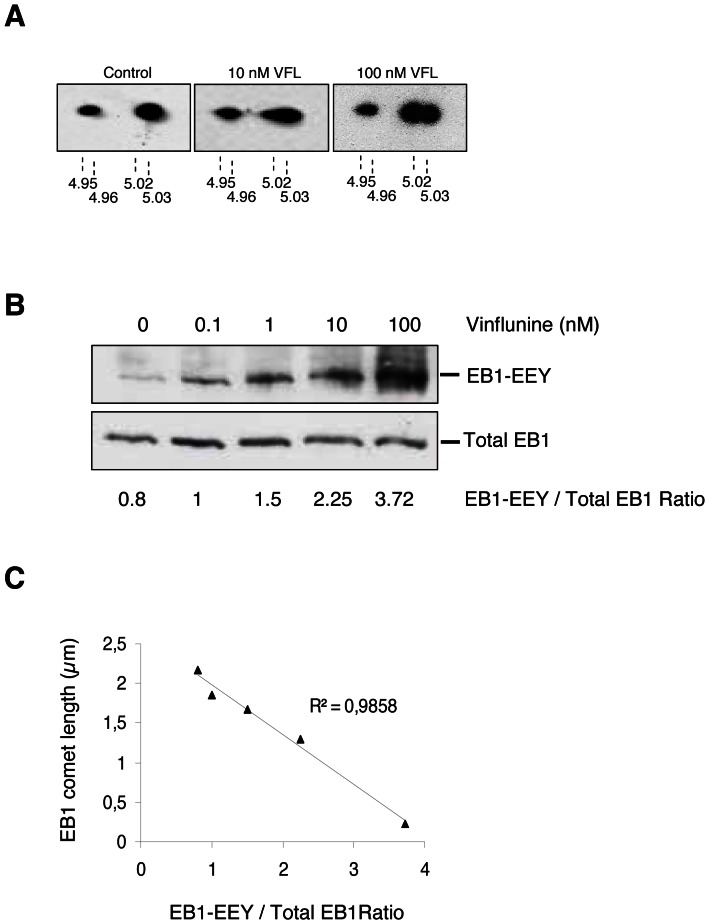
Anti-migratory vinflunine favored the tyrosinated form of EB1. (**A**) Effect of VFL on EB1 focalization on 2D gel electrophoresis. Cells were incubated for 5 h with VFL (10, 100 nM) or vehicle alone. Indicated experimental isoelectric points were calculated according to reference protein (stathmin) (**B**) Detection of EB1-EEY sequence with YL½ antibody increased in a concentration-dependent manner under VFL concentrations. Relative ratios are presented under the blots. (**C**) Correlation between detection of EB1-EEY sequence and reduction of EB1 comet length by VFL.

We then assessed whether VFL regulated EB1 at a post-translational level. In endothelial cells, VFL (10 nM) altered EB1 2D profile by increasing focalization at isoelectric point 5.02 (native tyrosinated form) with a concomitant decrease at the isoelectric point 5.03 (detyrosinated form) (Figure 5A). This effect was more pronounced with 100 nM VFL as the two spots were clearly visible.

Such dose-response effect was comforted by detection of the EB1-EEY epitope with YL½ antibody in 1D electrophoresis. Indeed, VFL increased the EB1-EEY/EB1 ratio, in a concentration-dependent manner (Figure 5B). Interestingly, this dose effect was correlated to the decrease in EB1 comet length induced by VFL (Figure 5C, correlation coefficient = 0.98).

**Figure 6:**
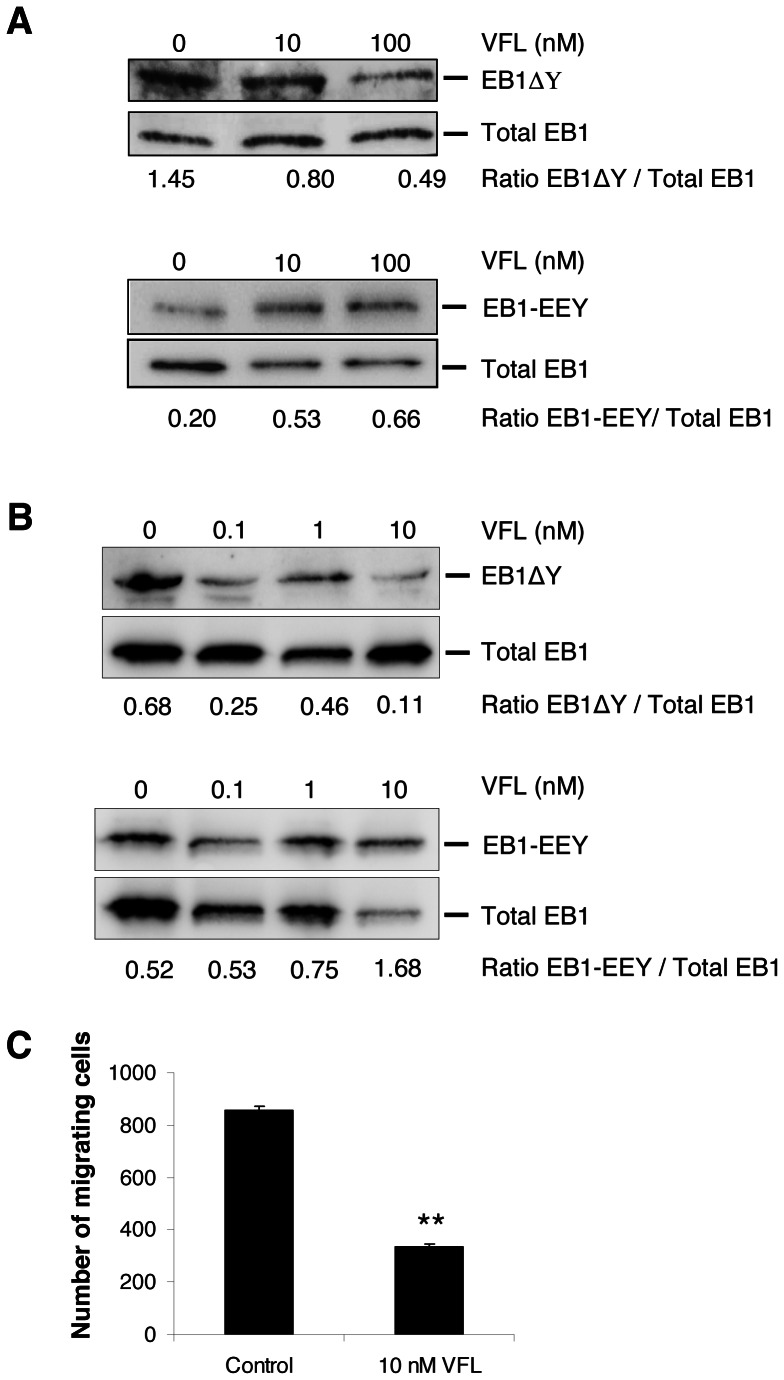
Anti-migratory vinflunine altered EB1 detyrosination/retyrosination cycle in both endothelial and glioblastoma cells. Western blotting detection of detyrosinated or tyrosinated EB1 in HUVECs (**A**) and U87 cells (**B**) under VFL treatment. Relative ratios detyrosinated EB1 (EB1ΔY)/total EB1 or tyrosinated EB1 (EB1-EEY)/total EB1, from at least five independent experiments, are presented under the blot. (**C**) Transwell migration assay with control sh0 U87cells treated or not with VFL (10 nM). Data show the average number of cells that migrated through the filter. At least three independent experiments were performed for each condition. *Bar* ± SEM (**) indicates significant differences from control (p<0.05).

We thus determined the effect of VFL on the equilibrium between tyrosinated and detyrosinated EB1 in both endothelial (Figure 6A) and glioblastoma cells (Figure 6B) by using specific antibodies [22]. As expected, according to the results obtained with YL½ in HUVECs, VFL increased the amount of tyrosinated EB1 while decreasing the amount of detyrosinated EB1 in a concentration-dependant manner, in both HUVECs and U87. Moreover, similarly to the results obtained in HUVECs at the concentration that induced the highest changes on EB1 tyrosination and detyrosination status, VFL inhibited glioblastoma cell migration (Figure 6C). Altogether, our results suggest that, in link with its anti-migratory potential, VFL interfered with EB1 tyrosination/detyrosination cycle.

## Discussion

### Inhibition of EB1 accumulation at MT (+) ends: a marker of MTA anti-migratory effect

Our previous works suggested a crucial role of MT dynamic instability and EB1 in angiogenesis and cell migration [20], [21]. Indeed, we demonstrated that the anti-migratory effect of MTAs, such as VFL and patupilone, was associated with alteration of EB1 comets and an increase in MT dynamic instability [16], [19], [20], [21]. By contrast, here we showed that the pro-angiogenic/pro-migratory VEGF increased EB1 accumulation at MT (+) ends without significant change in its expression level, induced EB1 to bind along the MT lattice, and suppressed MT dynamic instability in human endothelial cells. These data are consistent with the established role of both MT stabilization during cell migration [3], [6] and EB1 involvement as a key player in microtubule-cell cortex interaction [4], [20], [21], [33].

MT dynamic instability parameters and particularly MT overall dynamicity, which is known to be associated with the level of cell proliferation inhibition [14], [34] is not a relevant marker of MTA anti-migratory properties. Indeed, paclitaxel or VFL increased overall dynamicity at low concentrations in endothelial cells [16], [19], it was not modified by patupilone in glioblastoma cells [21] and decreased at high cytotoxic concentrations. On the other hand, EB1 appeared to be a more reliable marker as we found a strong correlation between EB1 accumulation at MT (+) ends and the inhibition of endothelial migration by VFL at all effective concentrations. These results are strengthened by recent works demonstrating that EB1 down-regulation impairs migration [35] and capillary-like tube formation in endothelial cells [36]. Taken together these observations make EB1 comets a crucial marker of cell migration and a potential target in neoangiogenesis.

### Changes in the −EEY sequence of EB1 was associated with EB1 comet length

By exploring the post-translational modifications that could be associated with EB1 comet changes under VEGF treatment, we found that the EB1 C-terminal −EEY sequence was weakly detectable in control cells and totally disappeared in VEGF-treated cells (Figure 2B) when using the YL ½ antibody that specifically recognizes the −EEY epitope [26]. This result suggests that a part of EB1 protein in endothelial cells, is not the native tyrosinated form and that this form is induced by VEGF. The alteration of the −EEY sequence of EB1 may affect its accumulation at MT (+) end as well as its functions in the regulation of MT dynamics. EB1 comet length is usually expected to be proportional to MT growth. However, VEGF increased EB1 accumulation at MT (+) end while suppressing MT growth rate in a counterintuitive manner. This result may be explained by the alteration of the −EEY sequence on EB1 and the consequent loss of p150^glued^ binding at MT (+) end in VEGF-treated cells. Indeed, the MT growth promoting activity of EB1 has been associated with its C-terminal interaction with CAP-Gly proteins such as p150^glued^ [37]. This interaction of EB1 with CAP-Gly domain containing proteins requires the −EEY sequence and particularly the last tyrosine residue [29], [38]. Elsewhere, it appeared that the GFP-EB1ΔY construct bound better to MT (+) end than GFP-EB1 full length (data not shown). Similar results have been reported for GFP-EB1-Δ7, a construct that lacks the C-terminal sequence [39]. The reasons for such differences between these constructs may be explained by the fact that the −EEY sequence is involved in EB1 auto-inhibition [37], [40]. Alternatively, EB1 C-terminus holds negative charges that can also inhibit its binding on the MT lattice [41].

### Modifications of the EB1-EEY sequence suggested a cycle of detyrosination/retyrosination

Due to the high similarity in the last amino acid sequence between tubulin and EB1, one can hypothesize that EB1-undergoes a C-terminal detyrosination a reversible modification present on tubulin [26], [31], [42]. As expected, the incubation of cells with 3-L-nitrotyrosine resulted in the incorporation of this non-genetically coded amino acid into tubulin. Such incorporation is mainly due to the cycle of detyrosination/retyrosination that occurs at the C-terminus of tubulin and is regulated by a carboxypeptidase and a specific tubulin tyrosine ligase (TTL) [43]. Interestingly, EB1 was also able to incorporate the non-genetically coded 3-L-nitrotyrosine amino acid suggesting that, as tubulin, EB1 may undergo a cycle of detyrosination/retyrosination. The existence of such cycle for EB1 is strengthened by the detection, thanks to specific antibodies, of both detyrosinated and tyrosinated EB1 in endothelial and glioblastoma cells; the opposite variation of the detyrosinated EB1 and the tyrosinated form under VFL treatment in both endothelial and glioblastoma cells; and the partial retyrosination of the GFP-EB1ΔY construct in glioblastoma U87 cells.

According to the existence of a detyrosination/retyrosination cycle of EB1, we detected nitrotyrosine on EB1 after EB1 immunoprecipitation in HUVECs incubated with VEGF suggesting that VEGF induced EB1 tyrosine nitration (data not shown). Protein nitrotyrosination is a physiological process that involves peroxynitrite, a highly reactive oxidant formed by the rapid combination of NO with superoxide anion. Interestingly, the production of NO by the endothelial Nitric Oxide Synthase (eNOS) is essential for VEGF-induced angiogenesis [44], [45] and inhibition of HUVECs migration by microtubule-targeted drugs is associated with disruption of VEGF-induced eNOS activation and NO production [18], [46]. The nitrotyrosinated form of EB1 may also account for the alteration of −EEY sequence in VEGF-treated cells.

### EB1 detyrosination in cell migration, tumor progression and effect of MTAs

Elevated levels of detyrosinated tubulin are known to be associated with cell migration [47]. In the last 10 years, important functions of tubulin tyrosination have been discovered. Thus, TTL loss and the resulting tubulin detyrosination confer selective advantage to cancer cells during tumor growth [48], [49], [50]. Recently, a role for detyrosinated tubulin in epithelial to mesenchymal transition, important in development, stem cell differentiation, and tumor invasion has been proposed [51]. The last tyrosine residue of α-tubulin C-terminal tail is crucial for the binding of CAP–Gly proteins [13] suggesting that the phenotypes observed after TTL suppression may arise from mislocalization of CAP-Gly proteins at detyrosinated MT (+) ends. However, the CAP-Gly (+) end tracking also involves EB proteins [7], [52] and we demonstrated that the last tyrosine residue is crucial for EB interaction with CAP-Gly proteins [29]. Thus, EB1 detyrosination may be a complementary and/or an alternative process to tubulin detyrosination. However, we cannot conclude about the involvement of tubulin regulating enzymes in EB1 cycle. AGBL2, a recently discovered tubulin carboxypeptidase that leads to tubulin detyrosination, and its inhibitor RARRES1, form a complex with EB1 [53] suggesting that AGBL2 could be a good candidate potentially involved in EB1 detyrosination. Nevertheless, our data clearly demonstrated that cells containing detyronisated EB1 had a higher migratory potential than cells containing the tyrosinated form. This result suggests a potential role of EB1 detyrosination in tumor progression. Interestingly, our data showed that MTAs such as VFL altered EB1 detyrosination/tyrosination cycle and favored tyrosinated EB1 in a concentration-dependant manner. Such results suggest that VFL, besides its known cellular effects on MT dynamics and EB1 accumulation at MT (+) ends, may also antagonize EB1 detyrosination process.

In conclusion, our study highlights the existence of EB1 detyrosination/tyrosination cycle that appears to be related to endothelial and glioblastoma cell migration. These findings open new avenues of research in basic knowledge of +TIPs regulation in cancer and to further evidence potential specific targets for cancer therapy.

## Supporting Information

Figure S1 **VEGF enhanced HUVECs migration.** (**A**) Random motility coefficient of HUVECs incubated with VEGF (1 h; 10 ng/ml) pre-incubated or not with VEGF trap (**B**) Recovered surface by HUVECs in wounding assay in control and VEGF treated cells. At least three independent experiments were performed for each condition. *Bar* ± SEM (* and **) indicates significant differences between conditions (p<0.05 and p<0.005 respectively).
(TIF)

Figure S2 **VEGF induced MT targeting to adhesion sites.** (A) Video frame of HUVECs transfected with GFP-tubulin visualized by fluorescence microscopy and by TIRF showing MT close to the basal membrane. Cells were either untreated or incubated for 1 h with VEGF (10 ng/ml) or VEGF Trap. *Bar*, 10 µm. (B) Video frame of HUVECs co-transfected with GFP-tubulin and DsRed-paxilin. Cells were either untreated or incubated for 1 h with VEGF (10 ng/ml) or VEGF Trap. *Bar*, 10 µm. (**C**) Quantification of percentage of MT (+) ends targeting adhesion site on HUVECs after treatment with VEGF (10 ng/ml) or VEGF-Trap. Bar ± SEM (*) indicates significant differences from control (p<0.05).
(TIF)

Figure S3 **EB1 focalized at different isoelectric points in endothelial and glioblastoma cells.** EB1 profiles from 2D gel electrophoresis of total human HUVECs and U87 glioblastoma cell lysates. Indicated experimental isoelectric points were calculated according to reference protein (stathmin).
(TIF)
