# Supplementary figures and images for: Anti-Migratory Effect of Vinflunine in Endothelial and Glioblastoma Cells Is Associated with Changes in EB1 C-Terminal Detyrosinated/Tyrosinated Status
Source: PLoS One. 2013 Jun 4;8(6):e65694. doi: 10.1371/journal.pone.0065694 (PMC3672205; doi:10.1371/journal.pone.0065694)

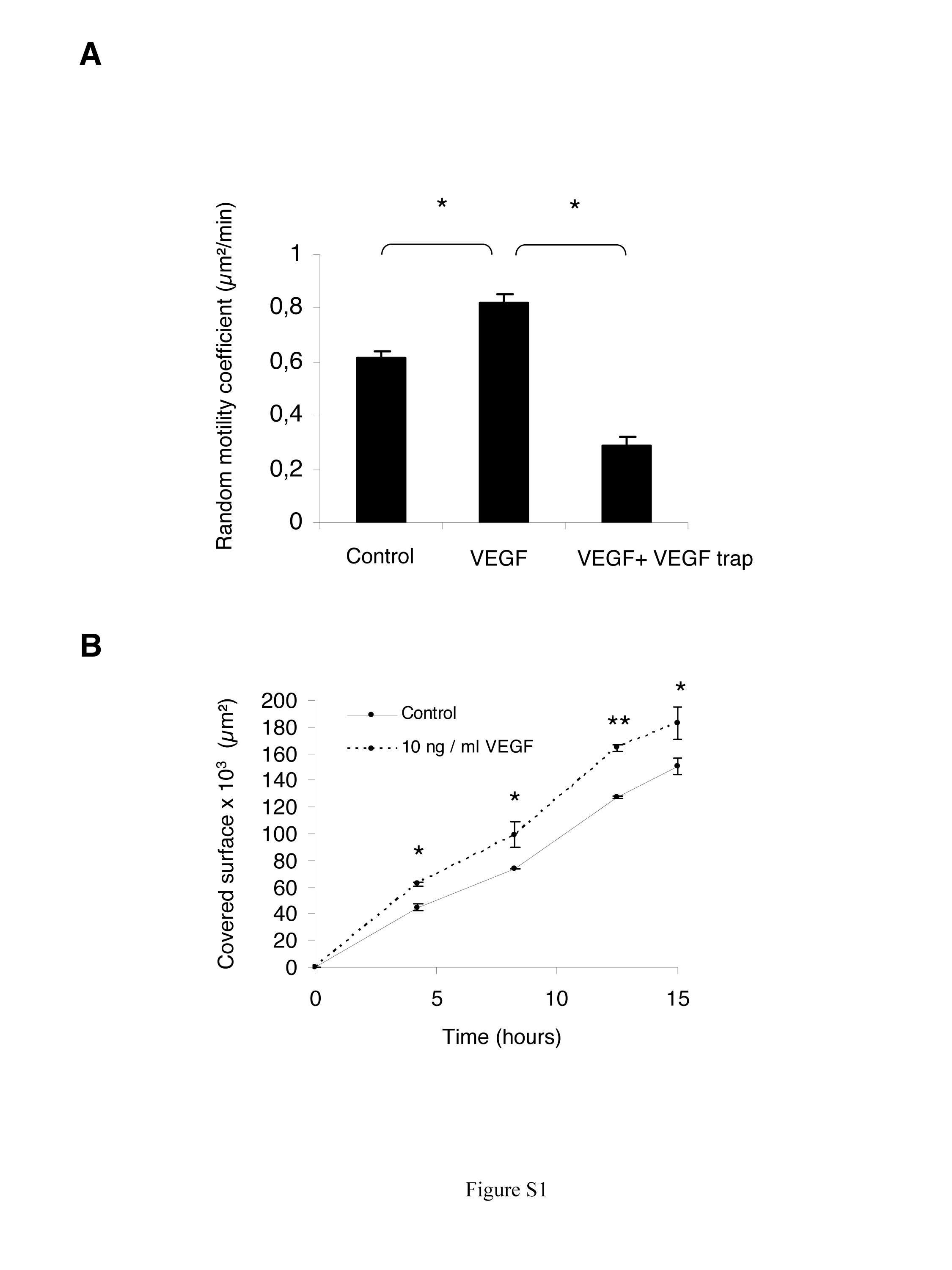

Supplement: Figure S1 — VEGF enhanced HUVECs migration. (A) Random motility coefficient of HUVECs incubated with VEGF (1 h; 10 ng/ml) pre-incubated or not with VEGF trap (B) Recovered surface by HUVECs in wounding assay in control and VEGF treated cells. At least three independent experiments were performed for each condition. Bar ± SEM (* and **) indicates significant differences between conditions (p<0.05 and p<0.005 respectively). (TIF) [file pone.0065694.s001.tif]

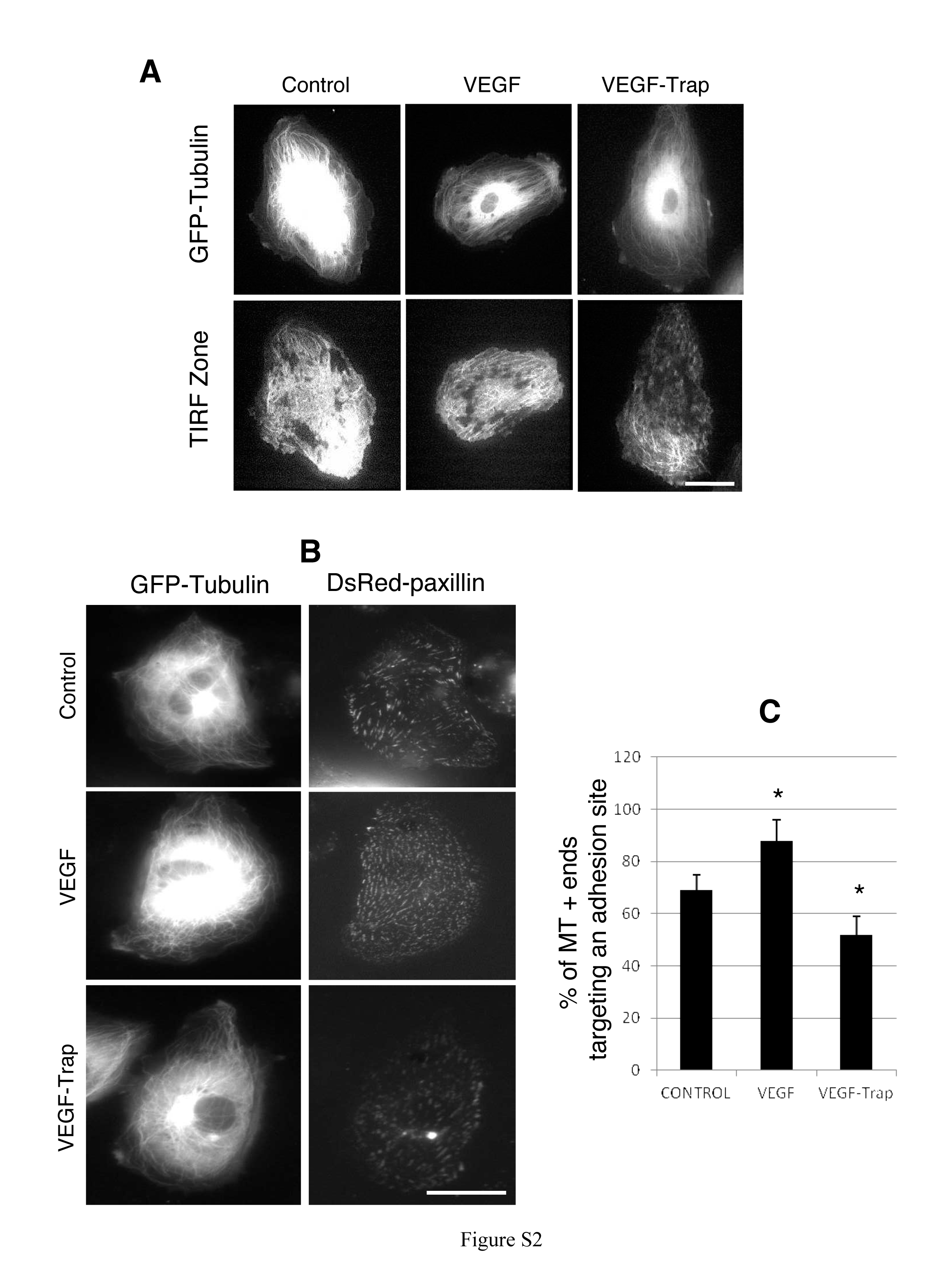

Supplement: Figure S2 — VEGF induced MT targeting to adhesion sites. (A) Video frame of HUVECs transfected with GFP-tubulin visualized by fluorescence microscopy and by TIRF showing MT close to the basal membrane. Cells were either untreated or incubated for 1 h with VEGF (10 ng/ml) or VEGF Trap. Bar, 10 µm. (B) Video frame of HUVECs co-transfected with GFP-tubulin and DsRed-paxilin. Cells were either untreated or incubated for 1 h with VEGF (10 ng/ml) or VEGF Trap. Bar, 10 µm. (C) Quantification of percentage of MT (+) ends targeting adhesion site on HUVECs after treatment with VEGF (10 ng/ml) or VEGF-Trap. Bar ± SEM (*) indicates significant differences from control (p<0.05). (TIF) [file pone.0065694.s002.tif]

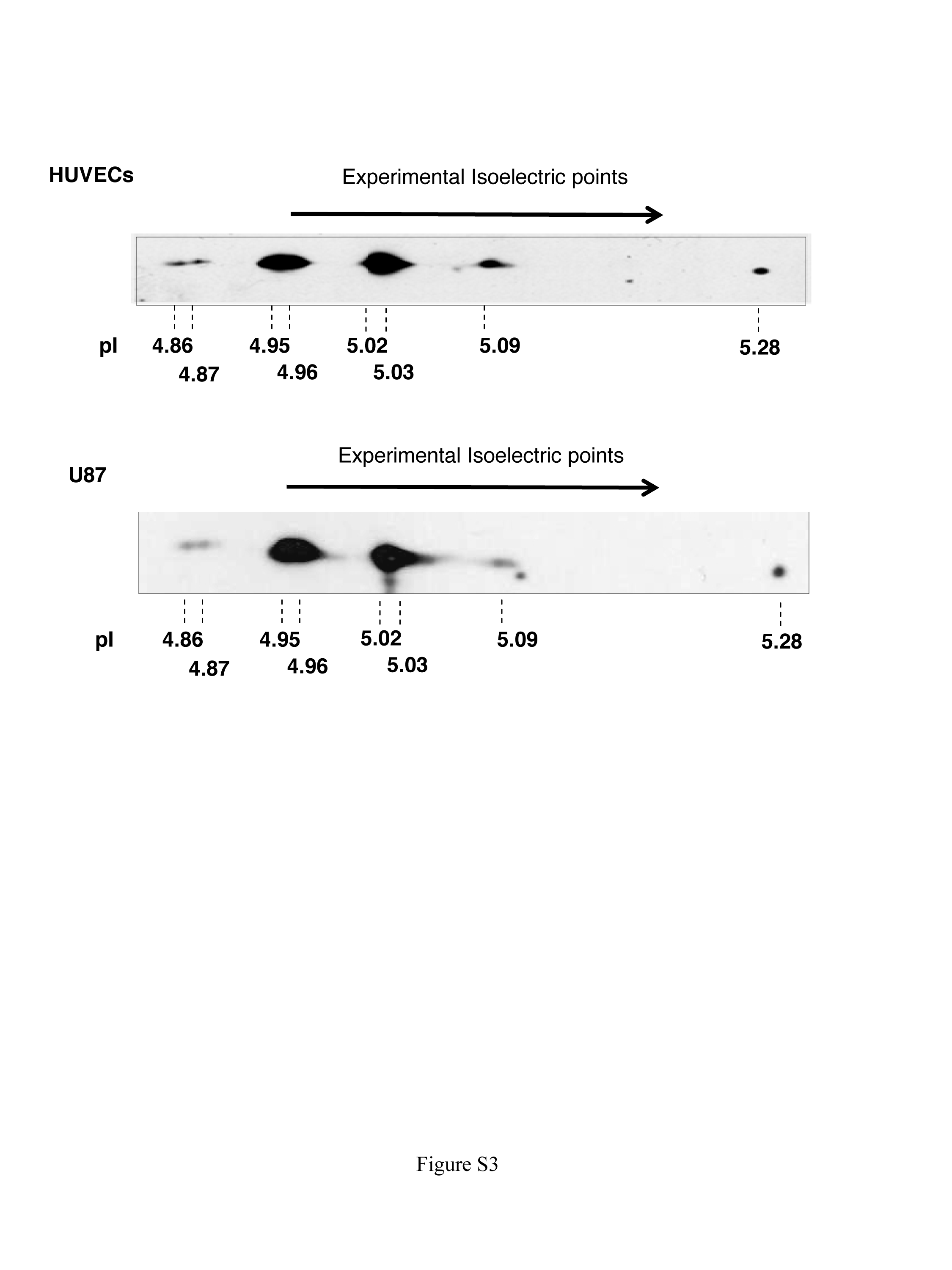

Supplement: Figure S3 — EB1 focalized at different isoelectric points in endothelial and glioblastoma cells. EB1 profiles from 2D gel electrophoresis of total human HUVECs and U87 glioblastoma cell lysates. Indicated experimental isoelectric points were calculated according to reference protein (stathmin). (TIF) [file pone.0065694.s003.tif]
